# Management of enzyme diversity in high-performance cellulolytic cocktails

**DOI:** 10.1186/s13068-017-0845-6

**Published:** 2017-06-19

**Authors:** Francisco Manuel Reyes-Sosa, Macarena López Morales, Ana Isabel Platero Gómez, Noelia Valbuena Crespo, Laura Sánchez Zamorano, Javier Rocha-Martín, Fernando P. Molina-Heredia, Bruno Díez García

**Affiliations:** 1 Department of Biotechnology, Abengoa Research, Campus Palmas Altas, C/Energía Solar 1, 41014 Seville, Spain; 2 Instituto de Bioquímica Vegetal y Fotosíntesis, Universidad de Sevilla y CSIC, Américo Vespucio 49, 41092 Seville, Spain

**Keywords:** Enzymatic hydrolysis, Cellulolytic cocktail improvement, Lignocellulosic biomass, Bioethanol

## Abstract

**Background:**

Modern biorefineries require enzymatic cocktails of improved efficiency to generate fermentable sugars from lignocellulosic biomass. Cellulolytic fungi, among other microorganisms, have demonstrated the highest potential in terms of enzymatic productivity, complexity and efficiency. On the other hand, under cellulolytic-inducing conditions, they often produce a considerable diversity of carbohydrate-active enzymes which allow them to adapt to changing environmental conditions. However, industrial conditions are fixed and adjusted to the optimum of the whole cocktail, resulting in underperformance of individual enzymes.

**Results:**

One of these cellulolytic cocktails from *Myceliophthora thermophila* has been analyzed here by means of LC–MS/MS. Pure GH6 family members detected have been characterized, confirming previous studies, and added to whole cocktails to compare their contribution in the hydrolysis of industrial substrates. Finally, independent deletions of two GH6 family members, as an example of the enzymatic diversity management, led to the development of a strain producing a more efficient cellulolytic cocktail.

**Conclusions:**

These data indicate that the deletion of noncontributive cellulases (here EG VI) can increase the cellulolytic efficiency of the cocktail, validating the management of cellulase diversity as a strategy to obtain improved fungal cellulolytic cocktails.

## Background

Increasing awareness about global warming during the last decade has promoted renewed efforts for the development of alternative sources of energy, such as the extraction of the chemical energy trapped in the polymers of lignocellulosic biomass to produce biofuels for transportation. Nevertheless, the release of sugars from the complex carbohydrates from agricultural and woody wastes has been hindered by the low availability of efficient and affordable cellulolytic enzyme cocktails [1]. Thus, research focused on reducing costs and increasing the yield of biofuel production processes requires maximizing the performance of enzyme cocktails used to release fermentable sugars from biomass [2–4].

It is well known that the complete conversion of cellulose and hemicellulose into monomeric sugars requires the combined action of different classes of enzymes because individual enzymes are only capable of partially digesting the polymers. A larger number of enzymes are required for digesting hemicellulose to monomeric sugars than those for cellulose, including enzymes with hydrolase and esterase activities [5]. In addition, a number of auxiliary enzymes have been discovered to play an important role in boosting the cellulolytic machinery, such as expansin-like swolenins [6–8] and polysaccharide monooxygenases (PMOs) [9–11] not only increasing the performance but also the complexity of the enzymatic cocktails [12, 13].

Cellulolytic microorganisms in general, and the industrially preferred filamentous fungi in particular, are able to produce a considerable diversity of hydrolytic enzymes, with tens or even hundreds of individual genes being expressed under cellulolytic-inducing conditions [14]. These enzymes often have overlapping or even redundant activities which allow the producing organism to adapt to changing environmental conditions [15–21]. With a focus on commercial preparations, in a first attempt to understand the substrate-specific gene regulation and response, the commercial cellulolytic fungus *Trichoderma reesei* has been subjected to “fingerprinting” analysis by high-resolution 2D gel electrophoresis [22]. Such analyses identified more than 40 proteins evidencing the complexity of the system.

**Fig. 1:**
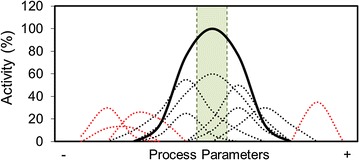
Graphic representation of enzymes and cocktail performance versus process conditions. *Solid line* represents the activity profile of the whole cocktail under a range of process conditions ( i.e., pH, temperature, substrate concentration, mixing, etc.). *Dotted lines* represent the activity profile for individual enzymes in the mixture. At industrially controlled conditions (*shadowed in green*) contributive enzymes (*black*) would be capable to act while less-contributive (*red*) would show a reduced or negligible activity

However, the industrial conditions for enzymatic hydrolysis of lignocellulosic biomass are not so variable. The process starts with a predigested (pretreated) material, where many acid and temperature-labile bonds are already broken. Those conditions are usually restricted to the optimal performance of the cocktail, usually around 50 °C and pH 5.0 [23]. Consequently, a high diversity of enzymes with overlapping activities might not be necessary to carry out the enzymatic hydrolysis at industrial scale [24]. A diverse and adaptable cellulolytic machinery has been considered till now as a measurement of the potential of the microorganisms and the cocktails. However, the extreme complexity of these cocktails and their flexibility is a key impediment to develop efficient artificial cellulase cocktails that the industry is demanding [25]. Furthermore, this diversity can be counterproductive since under industrial conditions only a fraction of the enzymes is able to contribute to the performance of the whole cocktail. Only those enzymes the optimal activity of which matches the operating conditions are effective (Fig. 1). The expression and subsequent production of redundant or activity-limited enzymes are therefore undesirable because it will dilute the presence of the best fitted, reducing the efficiency of the whole cocktail.

Kumar and Murthy [26] have recently summarized several experimental studies performed to determine the optimal enzyme cocktail for a specific feedstock by testing many combinations and ratios of a small number of pure enzymes from different organisms. Furthermore, other studies have shown that some combinations can even outperform the whole cocktails at low solids content [27], demonstrating that natural cocktails have the potential but are not optimized in terms of protein profiles and ratios for the specific materials and hydrolysis conditions. However, few examples have pursued this strategy to generate improved microorganisms that overexpress a low-diversity cellulolytic cocktail, with lower proportions or even eliminating “less-contributive” enzymes in favor of the most contributive ones. This higher efficiency will reduce the enzyme dose to obtain, at least, the same yield, improving the economy of the process.

In addition to this, Nevalainen and Peterson showed that the deletion of any secreted protein gene theoretically results in correspondingly higher yields of the rest of the secreted proteins [28]. In practice, this can be negligible if the deleted gene encodes a minor secreted protein, but it can be significant if the deleted gene corresponds to an abundant protein. Maintaining the titer of secreted proteins of the low-diversity cocktail under standardized production conditions could increase the efficiency of the cocktail without affecting the production cost. In the present work we demonstrate the improvement of the cocktail produced by *Myceliophthora thermophila* (formerly *Chrysosporium lucknowense*) [15, 29, 30] by deleting the abundant but less-contributive cellulase EG VI versus the also abundant but more-contributive CBH IIb as a proof of concept that removing less efficient cellulases from the commercial cocktails allows to improve the overall cellulolytic activity.

## Methods

### Strains and growth conditions

*Myceliophthora thermophila* industrial strain derived from C1 UV18-25 [30, 31] was obtained under license from Dyadic International Inc. (Jupiter, Florida). Strains were grown in shake flasks at 35 °C and 200 rpm for 5 days using culture media described by Emalfarb et al. [32]. The culture was centrifuged at 16,000×*g* during 40 min at 4 °C. The supernatant was further clarified with 0.45 µm nylon filters adding sodium acetate buffer (pH 5.0) to 50 mM. This extracellular enzyme solution is considered hereinafter as whole cocktail.

### LC–MS/MS analysis

All LC–MS/MS analyses were performed at the SCAI proteomics facility of the University of Córdoba (Spain). After clean up, the samples were reduced, alkylated and digested with trypsin using standard protocols. Briefly, nano LC was performed in Dionex Ultimate 3000 nano ultra-performance liquid chromatography (UPLC; Thermo Scientific) with an Acclaim Pepmap nanocolumn C18 75 μm × 150 mm, 3 μm particle size (Thermo Scientific). Previously, peptide mix was loaded in a 300 µm × 5 mm Acclaim Pepmap precolumn (Thermo Scientific) in 5% acetonitrile/0.1% formic acid for 5 min at 5 µl/min. Peptide separation was performed at 40 °C for all runs. Mobile phase buffer A was composed of water, 0.1% formic acid. Mobile phase B was composed of 80% acetonitrile, 0.1% formic acid. Samples were separated at 300 nl/min. Mobile phase B increased from 4 to 45% B for 60 min; 45–90% B for 1 min, followed by a wash of 5 min at 90% B and a 15 min re-equilibration at 4% B. Total time of chromatography was 85 min.

Mass spectrometry data (full scan) were acquired in the positive ion mode over the 400–1500 m/z range. Mass spectrometry data were acquired in a data-dependent scan mode, automatically selecting the five most intense ions for fragmentation. The Orbitrap resolution was set at 30,000, and dynamic exclusion was applied during 30-s intervals. The raw data was processed using Proteome Discoverer (version 1.4, Thermo Scientific). Mass spectrometry spectra were searched with SEQUEST engine against *Myceliophthora* or *Thielavia* genus [33, 34]. Peptides were generated from a tryptic digestion with up to one missed cleavages, carbamidomethylation of cysteines as fixed modifications, and oxidation of methionines as variable modifications. Other parameters like 10 ppm precursor mass tolerance and 0.8 Da product ion mass tolerance were used. Peptide spectral matches (PSM) were validated using percolator based on *q* values at a 1% FDR, against decoy database. With Proteome Discoverer software, peptide identifications were grouped into proteins according to the law of parsimony and filtered to 1% FDR.

### Enzymes purification

A 20 ml sample of the whole cocktail obtained with the procedure described above was loaded onto an SP-Sepharose column (GE Healthcare) equilibrated with the same buffer (50 mM sodium acetate, pH 5.0). EG VI was eluted with 1 M sodium chloride, 50 mM sodium acetate buffer (pH 5.0). Collected fractions were desalted using HiPrep 26/10 column (GE Healthcare) and were analyzed by mass spectrometry and SDS-PAGE to identify and check their purity. CBH IIa and CBH IIb were purified following the procedures described by Bukhtojarov et al. [35] and Gusakov et al. [27] respectively.

### Sequence analysis

The sequence homology between the EG VI protein and other known *M. thermophila* proteins was analyzed using the BLASP network service of the National Center for Biotechnology Information [36, 37]. The Signal peptides were analyzed with the SignalP 4.1 Server [38]. Mature protein sequence was analyzed using ProtParam [39].

### Enzymes characterization

Enzyme activity was tested with colorimetric methods widely used in the field. To standardize them, all the activities were measured at 50 °C in sodium acetate buffer pH 5.0 (with appropriate amounts of substrate and enzyme recommended by the manufacturer). The reaction time was fixed to 10 min, except avicellase activity that was run for 2 h of reaction time. Other reaction conditions are summarized in the Table 1. For all glycosyl hydrolase assays, one unit of activity (U) was defined as the amount of enzyme that converts one micromole of substrate or releasing one micromole of sugars (in glucose equivalents) per min. Protein concentration was determined by the BCA method with serum albumin as the standard.

**Table 1:** Enzymatic assays conditions

Activity	Substrate	Product reference	Substrate concentration (g/l)	Temp. (°C)	pH	Time (min)	References
Beta-glucosidase	pNPG^a^	Sigma N7006	0.1	50	5.0	10	[40]
Beta-xylosidase	pNXP^b^	Sigma 487870	0.1	50	5.0	10	[40]
Cellobiohydrolase	Avicel	Sigma 11365	10.0	50	5.0	120	[41, 42]
Endoglucanase	Azo-CMC^c^	Sigma 18693	10.0	50	5.0	10	[43]
Endoxylanase	Azo-WAX^d^	Megazyme	10.0	50	5.0	10	[44]

^a^ *p*-Nitrophenyl beta-d-glucopyranoside

^b^ *p*-Nitrophenyl-beta-d-xylopyranoside

^c^Azo-carboxymethyl cellulose

^d^Azo-wheat arabinoxylan

The optimal pH and temperature for the enzymatic activities were obtained using pure enzymes under the reaction conditions shown in Table 1 with Azo-CMC as substrate. For the thermostability analysis, pure enzyme solutions were incubated at 50 °C for up to 24 h and analyzed in parallel with untreated samples by gel electrophoresis (SDS-PAGE) under standard denaturing conditions. Images were captured by scanning Coomassie Brilliant Blue-stained gels using a GS-800 imaging densitometer (Bio-Rad) and were digitized with Multi Analyst software (Bio-Rad).

### Gene deletion

The deletion of *egVI* and *cbhIIb* genes was carried out using *amdS* gene cassettes encoding acetamidase as a reversible marker [45]. Two deletion plasmids were constructed flanking *amdS* gene cassette with upstream and downstream fragments of the *egVI* and *cbhIIb* genes respectively, to replace endogenous genes by the linearized *amdS* constructs by double homologous recombination.

Upstream fragments of *egVI* (2005 bp) and *cbhIIb* (1459 bp) genes were amplified using genomic DNA of *M. thermophila* extracted with DNeasy Plant Mini Kit (Qiagen). DNA sequences of used oligonucleotides are shown in the Table 2. PCR amplifications were carried out using iProof High-Fidelity DNA polymerase (Bio-Rad) and two primers (1 and 2 for *egVI*; 3 and 4 for *cbhIIb*) designed with restriction sites recognizable by enzymatic tandem SacI-BamHI (for *egVI*) and NotI-SmaI (for *cbhIIb*). The same strategy was followed to amplify the downstream sequence (2018 bp) of *egVI* and (1591 bp) *cbhIIb* using two different oligonucleotides (5 and 6 for *egVI*; 7 and 8 for *cbhIIb*) that included recognition sites for EcoRI-XhoI enzymes for both genes. PCR conditions were optimized to 95 °C during 2 min followed by 30 cycles of 98 °C during 10 s, 55 °C 20 min, 72 °C 90 s and 72 °C during 10 min.

**Table 2:** Oligonucleotides used

References	DNA sequence
[1]	ACCGAGCTCGTAGCACTCGCTGTGTATCCTC
[2]	CCTGGATCCCTTATACCCAGGACATTCACAGTTC
[3]	AGCTCCACCGCGGTGGCGGCCGCGATTAACAGGCTTGTTAAAGGAAGTCTTCACG
[4]	TAGGTTAGAGCTGCAGCCCGGGGAAACAAGCAACTATCTCGGGGCGGGA
[5]	ACCGAATTCATCAAATGGATAGGTCGGTAATG
[6]	CACCTCGAGCAAGGAAGTCGAGTACGAGTCC
[7]	CATGGTCATAGAATTCGATATCCATGGGCCTGATTGGGTTCATTGACCATG
[8]	GGGTACCGGGCCCCCCCTCGAGACATGGGCGCCCTCTTTAGTGGTGGACTTA
[9]	GGCTCGAGATCTACAAGACTG
[10]	GTAGTTGGACACGTTGGTGA
[11]	CCTACACGCCCAATGCTCGAGCTTGCTC
[12]	TCCGTCCAATCAGAGTGGAACGAATCAACA

Both fragments were cloned into a plasmid containing *amdS* gene that allows transformed cells to grow on acetamide as sole nitrogen source. To carry out this cloning, the downstream fragments and the plasmid digested with the enzymes described above were subject to ligation and transformed into *Escherichia coli* XL1-Blue MRF cells following the protocol described by the supplier (Stratagene). The resulting constructs were used to clone the upstream fragments with a similar procedure using the restriction enzymes sites included in the primers.

Deletion plasmids linearized with SacI and KpnI enzymes were used to transform *M. thermophila* C1 protoplasts as described by Verdoes et al. [31] and the patented procedure [46]. The product of each transformation was spread on agar petri dishes containing 0.6 g/l of Acetamide (Merck). After 5 days of incubation at 35 °C the genomic DNA of growing colonies (expressing *amdS* gene) was extracted using DNeasy Plant Mini Kit. The deletion was confirmed with the amplification of an internal fragment of the gene. Oligonucleotides 9 and 10 were used to check *egVI* deletion and the pair 11 and 12 for *cbhIIb*. Amplification reactions were run following a cycle of 95 °C during 2 min, 30 cycles of 95 °C during 30 s, 55 °C 30 s, 72 °C 30 s and a final step of 72 °C during 10 min. The resulting amplification mix was analyzed by agarose gel compared with the same product of parental colonies (not transformed).

### Enzymatic hydrolysis

Pretreated corn stover (from now on, PCS) was prepared by steam explosion with diluted sulfuric acid at the Abengoa Bioenergy Biomass Pilot Plant in York, Nebraska, USA, following the procedure described by Alcántara et al. [24]. Hydrolysis of PCS (20 g) was performed in 100 ml borosilicate glass bottles with airtight screw caps. Water was added to adjust the solid loading to 20% of total solids. The pH was initially adjusted to 5.5 by addition of NH_4_OH and no additional buffer was used to reproduce industrial hydrolysis conditions. The final enzyme loading was 10 mg of total protein per g of glucan. For the enzyme supplementation experiments over whole cocktails a final dose of 10 mg of whole cocktail plus 2 mg of pure protein per g of glucan was used. Glucan content was determined according to the standard biomass analytical procedures by NREL [47]. The hydrolysis was incubated at 50 °C under orbital shaking at 150 rpm for 72 h. Samples were taken at *t* = 0 and *t* = 72 h of hydrolysis and were processed for analysis according to Kristensen et al. [48]. Due to the high density of the hydrolysate at 20% of solids, the analytes were quantified in weight/weight (g/kg).

### Sugar analysis

After enzymatic hydrolysis, samples were filtered and analyzed by high performance liquid chromatography (HPLC) using an Aminex column HPX-87H of 300 mm × 7.8 mm with 9 µm particle size (Bio-Rad, California, USA). The analyses were performed at 60 °C under isocratic conditions with 5 mM H_2_SO_4_ as mobile phase at a flow rate of 0.6 ml/min with 20 µl injection volume. Carbohydrates (glucose, xylose and arabinose) were analyzed using a refractive index detector.

## Results and discussion

### Composition of whole cocktails

LC–MS/MS yielded a total of 202 different peptides matching proteins as indicated in the ‘‘Methods” section. A total of 79 proteins were identified using Mascot but almost 40% of them remained uncharacterized in the Uniprot database [33]. Some of them did not have detectable signal peptide using SignalP predictions or even could have a cytosolic or transmembrane subcellular location according with databases. The 27 proteins which are clearly recognized as extracellular glycosyl hydrolases are listed in Table 3.

**Table 3:** Carbohydrate-active enzymes identified

Main activity	Uniprot reference	Coverage^a^	GH family	Estimated % mol^b^
Beta-glucosidases/beta-xylosidases	G2QCQ3	40.7	3	21.2
	G2QDN2	1.2		0.3
Endoglucanases	H2B658	27.5	5	3.0
Endoglucanases/type II cellobiohydrolases	G2Q998 (EG VI)	42.8	6	7.5
	G2QFW6 (CBH IIa)	7.6		0.7
	G2QA39 (CBH IIb)	37.1		7.1
Type I cellobiohydrolases	G2Q665	13.1	7	6.3
	G2QCS4	2.3		0.3
	G2QGA1	12.7		1.3
	G2QNN8	3.8		0.3
	G2Q359	7.0		0.7
Endoxylanases	G2QJ91	14.0	10	2.0
Endoglucanases/xyloglucanases	G2QKQ0	13.0	12	0.3
Wide diversity	G2QHP5	9.6	16	1.0
	G2QLD1	17.1		1.7
Chitinases/beta-*N*-acetyl-glucosaminidases	G2QGV8	1.1	18	0.3
Endoglucanases	G2Q0Y0	5.3	45	0.3
Polysaccharide monooxygenases	G2Q4M0	13.5	61^c^	0.7
	G2Q7A5	15.8		0.7
	G2Q9F7	20.9		3.0
	G2Q9T3	9.9		0.7
	G2QAB5	37.2		3.8
	G2QCJ3	37.1		13.7
	G2QNT0	39.1		2.0
Alpha-glucanases	G2QMP5	1.9	71	0.3
Xyloglucanases	G2QHR7	16.4	74	2.0
Arabinobiosidases	G2QJ26	3.3	93	0.3

^a^Percentage of the protein sequence covered by identified peptides

^b^Relative quantification by exponentially modified protein abundance index (emPAI)

^c^Formerly GH61 and later reclassified as AA9

Several proteomic approaches have been followed to quantify the relative amount of each individual protein in the mixture, such as the quantification of the peptide abundance in MALDI by exponentially modified protein abundance index (emPAI) [49] shown in the Table 3. It has to be considered that protein profile may change depending on growth media and cultivation conditions [22, 50]. The glycosyl hydrolases identified represented more than 81.4% of the total cocktail while non carbohydrate-active enzymes (52 proteins) accounted for the remaining 18.6%.

In spite that *M. thermophila* has been reported to produce a highly diverse cellulase cocktail, the analysis reported here showed that in the C1 derived industrial strain the cocktail is not so diverse, containing just about two dozens of enzymes belonging to a few GH families. On the other hand, these analyses did not show the diversity of functional isoforms that post-translational modifications like glycosylation can introduce in the sample. Relatively low coverage values could be indicative of high rate of peptide mass modifications with respect to their theoretical mass. Proteins are identified by their aminoacid sequence and maybe several protein isoforms should be expected as described for many fungal secreted proteins [51–53].

Polysaccharide monooxygenases (formerly GH61 and later reclassified as AA9) was the most abundant family in terms of entries in this cocktail, followed by glycosyl hydrolases families 6 and 7. The important contribution of PMOs has been profusely demonstrated in the last years [9–11]. However, in terms of relative abundance, AA9 family, represented mainly by G2QCJ3, was the second family after GH3, which was represented principally by the beta-glucosidase G2QCQ3 as the most abundant protein in the cocktail. This enzyme is required for the final release of glucose from cellobiose and higher oligomers. On the other hand, two GH6 family members, G2Q998 and G2QA397, formerly named as EG VI and CBH IIb, shared the third position in relative abundance. GH6 family includes endoglucanases and cellobiohydrolases which perform catalysis with inversion of anomeric stereochemistry. Endoglucanases act on amorphous regions of cellulose to create engaging and releasing points for the processive action of cellobiohydrolases (CBHs). CBHs from family 6 act from the nonreducing ends of cellulose chains to generate cellobiose, while enzymes from family 7 act from reducing ends. These enzymes are, equally recognized as key components in the multienzyme cellulase complexes, being responsible for most of the solubilization of cellulose to oligomers and soluble sugars [54]. These enzymes are among the best characterized cellulases, including studies about several CBHs from *M. thermophila* [27, 35, 41]. These previous works also demonstrated that a simple cocktail with only a few purified enzymes could be more efficient than a more diverse enzyme mixture, including enzymes with a residual contribution to the overall sugar release.

The key consideration here is that for a given enzyme cocktail dose, which is the standard parameter to measure the economic performance, the elimination of the less-contributive enzymes increases the abundance of the most active ones. Obviously, for a cellulolytic cocktail the deletion of non carbohydrate-active proteins has been prioritized, although 52 proteins with a very low abundance still remain.

Based on the picture of cocktail diversity obtained in Table 3, candidates of the GH6 family representing about 15% of the protein content in the cocktail were selected for analysis and as a proof of concept of cocktail improvement by reduction of cellulase diversity.

### Benchmarking of GH6 enzymes

The three identified proteins belonging to the GH6 family exhibit a low percentage of identity among them, EG VI exhibits a 41% compared with CBH IIa, 38% with CBH IIb, and these last two share a 51% between themselves, suggesting their activities and/or substrate specificities are different. Only CBH IIb contains a cellulose-binding module (CBM) in its sequence. These enzymes were initially purified and characterized by Bukhtojarov et al. [35] and Gusakov et al. [27], showing that EG VI has predominantly endoglucanase activity whereas CBH IIa and CBH IIb are cellobiohydrolases.

In order to further characterize these enzymes to select the best candidate for deletion, pure preparations were obtained and analyzed by SDS-PAGE. Pure enzymes showed an apparent molecular weight slightly higher than the theoretical deduced form their sequences without signal peptide (~47 kDa instead of 39.4 kDa for EG VI; ~43 kDa instead of 40.6 kDa for CBH IIa and ~70 kDa instead of 49.4 kDa in the case of CBH IIb). These differences could be explained by the fact that some fungal glycosyl hydrolases are frequently modified post-translationally harboring *O*- and/or *N*-glycans [51–53].

Purified proteins were tested on various substrates under the standard assay conditions (see Table 1). As shown in Table 4, the three enzymes hydrolyzed Azo-CMC substrate, although EG VI activity was somewhat ten times higher. The activity against Avicel was higher for CBH IIb. Azo-WAX activity was five and ten times higher also for EG VI compared with CBH IIa and CBH IIb respectively. Activities on chromogenic substrates pNGP and pNXP were not detected with any of the enzymes. These results were in agreement with previous reports obtained with these enzymes [27, 35, 46].

**Table 4:** Characterization of purified GH6 enzymes

Enzyme	Mol. mass (kDa)	CBM presence	Optimal temperature & pH	Thermostability^a^	Azo-CMC (U/g)	Azo-WAX (U/g)	Avicel (U/g)
G2Q998 (EG VI)	47	No	65 °C; 5.5–6.0	Unstable	5492.1	1335.6	89.7
G2QFW6 (CBH IIa)	43	No	60 °C; 4.5	Stable	431.5	207.2	102.5
G2QA39 (CBH IIb)	70	Yes	60 °C; 5.0	Stable	560.0	108.0	192.6

^a^Stable/unstable means presence/absence of the protein band in SDS-PAGE after incubation at pH 5.0 and 50 °C during 24 h

The characterization shown in Table 4 resulted in optimal temperature and thermostability, as well as pH in agreement with previous data reported for these proteins [23]. The optimal temperature of EG VI was also slightly higher than for the other GH6 enzymes. However the protein band corresponding to the enzyme disappeared after being incubated at pH 5.0 and 50 °C during 24 h. According Bukhtojarov et al. after 5 h incubation at pH 5.0 EG VI lost 40% of its initial activity [35]. On the other hand, CBH IIa and CBH IIb conserved respectively more than 70 and 100% of their initial activity under the same conditions [27, 46]. Optimal pH value of EG VI (5.5–6.0) and CBH IIa (4.5) did not match the standard 5.0 for industrial hydrolysis which could also limit the contribution of these enzymes in the overall process.

In any case, the most relevant difference could be the stability of the enzymes, because the data on activity over commonly used model substrates is usually not very representative of the real activity on the pretreated biomass under industrial reaction conditions.

Much of the difference comes from the high solids loading used in the industrial reactions. Large solids content give rise to strong solute interactions and inhibition of the enzymes due to higher sugar concentrations. These conditions are not at all comparable to the measurements of activity with pure enzymes on model substrates. In the end, the only way to determine the contribution of each enzyme to the hydrolysis is the individual supplementation of the whole cocktail with them under conditions reproducing the industrial reaction.

### Whole cocktail supplementations

**Fig. 2:**
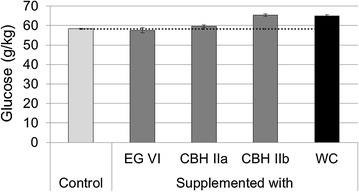
Glucose released by GH6 enzymes supplemented over whole cocktail (WC). Control (*light gray*): 10 mg/g of WC. EG VI (*dark gray*): 10 mg/g of WC supplemented with 2 mg/g of EG VI. CBH IIa (*dark gray*): 10 mg/g of WC supplemented with 2 mg/g of CBH IIa. CBH IIb (*dark gray*): 10 mg/g of WC supplemented with 2 mg/g of CBH IIb. WC (*black*): 12 mg/g of WC

The contribution to the enzymatic hydrolysis of the three purified GH6 enzymes was evaluated supplementing whole cocktails of *M. thermophila* C1 (Fig. 2). An enzyme is classified as contributive when it is able to release at least the same amount of glucose as that of an equal dose of the whole cocktail. An enzyme that yields less than the whole cocktail at the same dose is considered as noncontributive.

The addition of EG VI to a reaction with the control cocktail did not result in an increase of the release of glucose after 72 h. This could mean that EG VI could be in excess in the control cocktail or by the fact that this enzyme does not contribute (or relatively less than the whole cocktail) to the final cocktail performance under the assay conditions. A similar nonsignificant difference was obtained using CBH IIa, which suggests this is also a noncontributive enzyme. However CBH IIb addition released approximately the same glucose as the supplementation with the whole cocktail. This indicates that CBH IIb is a contributive enzyme that could replace the same amount of the whole cocktail. Possible causes that could explain the differences in contribution between both CBHs would be the lower optimal pH of CBH IIa and the presence of CBM in CBH IIb. These findings are in agreement with the results obtained with Avicel and native crystalline cellulose (cotton) at 40 °C by Gusakov et al. [27] where CBH IIb exhibited the highest hydrolysis rate and CBH IIa was notably less effective.

This kind of supplementation experiments is a very helpful tool to improve the enzyme cocktail and can help to predict the probable outcome of the overexpression of any enzyme of interest. We could expect that EG VI overexpression (even surpassing 16.6% of the whole cocktail, including the enzyme already present in the control cocktail plus the pure added one) would produce a cocktail releasing less glucose than the cocktail from the parental strain. On the other hand, a cocktail produced by a strain overexpressing CBH IIb would perform similar than the parental one. However, the synergism between cellulases is a well-known phenomenon that could affect this strategy, making the responses nonlinear, and forcing to test each dose/response empirically [12, 55, 56]. Multienzyme experiments at pH 5.0, 40 °C for 140 h performed by Gusakov et al. [27] have demonstrated that EG VI and CBH IIb enzymes act synergistically to hydrolyze cotton cellulose. This synergism was also evidenced with other cellulases present in this cocktail. Also parameters like a different cocktail composition, dosing, process conditions and the specific substrate have to be considered for the definition of the contributive or noncontributive enzymes. Altering any of these factors could change the picture.

Based on these results and considering the high abundance of the noncontributive enzyme EG VI, *egVI* gene was deleted to enrich the remaining contributive enzymes in the cocktail. The hydrolysis results are compared to the ones obtained by removing a contributive enzyme (CBH IIb).

### Performance of *ΔegVI*- and *ΔcbhIIb*-deleted enzyme cocktails

**Fig. 3:**
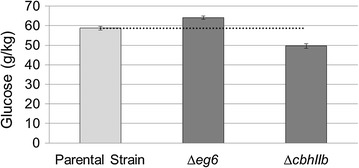
Glucose released after 72 h of hydrolysis of PCS by 10 mg/g of cocktails produced by Δ*egVI* and *ΔcbhIIb* strains compared with parental strain

The genes encoding *egVI* and *cbhIIb* were deleted independently to test the performance effects of deleting a contributive and a noncontributive enzyme gene. After the transformation with the linearized deletion constructs, the transformants were analyzed to verify if *egVI* or *cbhIIb* genes were substituted by the *amd*S gene. Strains with negative amplification of a 350 bp or 540 bp DNA fragments corresponding, respectively, to internal fragments of *egVI* or *cbhIIb* genes were selected and purified microbiologically by re-isolation prior to the production of the enzyme cocktails. Fermentable sugars released after enzymatic hydrolysis of PCS using these cocktails were compared with the cocktail produced by the parental strain (Fig. 3). After the enzymatic hydrolysis reactions, xylose and arabinose released were comparable (data not shown), while cocktails produced by deleted strains rendered different amounts of glucose compared with the parental strain. A cocktail that contains EG VI but not CBH IIb (secreted by *ΔcbhIIb* strain) released significantly less glucose than the parental strain, confirming the adscription of CBH IIb to the contributive class, while the cocktail containing CBH IIb but lacking of EG VI (produced by *ΔegVI* strain) resulted in a 10% increase of glucose release. This result suggests that the deleted enzyme EG VI was correctly identified as noncontributive, resulting in the production of a more efficient cocktail.

Finally, the success of managing cellulase diversity is strongly dependent not only on each enzyme cocktail but also on the hydrolysis conditions and the target substrate. We have validated this strategy using *M*. *thermophila* cocktail case by case against other substrates (like pretreated sugar cane straw), with other families of glycosyl hydrolases, i.e., GH7 or AA9, and under different reaction conditions (data not shown) proving that reduced cellulase diversity cocktails which maintain most contributive enzymes to the detriment of less-contributive ones is a successful strategy for the development of fungal multienzymatic cocktails.

## Conclusions

The aim of the present study was to demonstrate that the high diversity of enzymes produced by most cellulolytic fungi can be detrimental for an efficient industrial cocktail. For the design of an efficient cocktail, it is necessary to consider both the abundance and the performance of the individual enzymes on the real substrate. In this study, the cellulolytic cocktail produced by *M. thermophila*, as an example of a diverse and industrially recognized mix, was studied using LC–MS/MS. In spite of the high genomic potential described previously, only 27 carbohydrate degradation proteins were found. GH6 family was selected as a proof of concept, having only three members of relative abundance. The benchmarking of the enzymes was carried out by supplementation of the starting cocktail with the enzymes under question, using the industrial substrate and hydrolysis conditions. The comparison with the whole cocktail supplementation allowed us to classify the enzyme components as contributive or noncontributive. Using this criterion, the deletion of noncontributive cellulase EG VI led to the increase of the cellulolytic efficiency of the cocktail. This demonstrates the potential of the management of the enzyme diversity for the improvement of industrial cellulolytic cocktails.

## Abbreviations

Azo-CMC: azo-carboxymethyl cellulose
CBHs: cellobiohydrolases
CBM: cellulose-binding module
emPAI: exponentially modified protein abundance index
HPLC: high-performance liquid chromatography
LC–MS/MS: liquid chromatography tandem–mass spectrometry
NREL: National Renewable Energy Laboratory
PCS: pretreated corn stover
PMOs: polysaccharide monooxygenases
pNPG: *p*-Nitrophenyl beta-d-glucopyranoside
pNXP: *p*-Nitrophenyl-beta-d-xylopyranoside
UPLC: ultra-performance liquid chromatography

## References

[1] Álvarez C, Reyes-Sosa FM, Díez B (2016). Enzymatic hydrolysis of biomass from wood. Microb Biotechnol..

[2] Himmel ME, Ruth MF, Wyman CE (1999). Cellulase for commodity products from cellulosic biomass. Curr Opin Biotech..

[3] Galbe M, Zacchi G (2002). A review of the production of ethanol from softwood. Appl Microbiol Biotechnol.

[4] Nieves RA, Ehrman CI, Adney WS, Elander RT, Himmel ME (1998). Technical communication: survey and analysis of commercial cellulase preparation suitable for biomass conversion to ethanol. World J Microbiol Biotechnol.

[5] Van Den Brink J, De Vries RP (2011). Fungal enzyme sets for plant polysaccharide degradation. Appl Microbiol Biotechnol.

[6] Gourlay K, Hu J, Arantes V, Andberg M, Saloheimo M, Penttilä M, Saddler J (2013). Swollenin aids in the amorphogenesis step during the enzymatic hydrolysis of pretreated biomass. Bioresour Technol.

[7] Saloheimo M, Paloheimo M, Hakola S, Pere J, Swanson B, Nyyssönen E, Bhatia A, Ward M, Penttilä M (2002). Swollenin, a *Trichoderma reesei* protein with sequence similarity to the plant expansins, exhibits disruption activity on cellulosic materials. Eur J Biochem.

[8] Liu X, Ma Y, Zhang M (2015). Research advances in expansins and expansion-like proteins involved in lignocellulose degradation. Biotechnol Lett.

[9] Žifčáková L, Baldrian P (2012). Fungal polysaccharide monooxygenases: new players in the decomposition of cellulose. Fungal Ecol..

[10] Horn S, Vaaje-Kolstad G, Westereng B, Eijsink VG (2012). Novel enzymes for the degradation of cellulose. Biotechnol Biofuels.

[11] Dimarogona M, Topakas E, Christakopoulos P (2012). Cellulose degradation by oxidative enzymes. Comput Struct Biotechnol J..

[12] Woodward J (1991). Synergism in cellulase systems. Bioresour Technol.

[13] Gonçalves GAL, Takasugi Y, Jia L, Mori Y, Noda S, Tanaka T, Ichinose H, Kamiya N (2015). Synergistic effect and application of xylanases as accessory enzymes to enhance the hydrolysis of pretreated bagasse. Enzyme Microb Technol.

[14] Glass NL, Schmoll M, Cate JHD, Coradetti S (2013). Plant cell wall deconstruction by ascomycete fungi. Annu Rev Microbiol.

[15] Berka RM, Grigoriev IV, Otillar R, Salamov A, Grimwood J, Reid I, Ishmael N, John T, Darmond C, Moisan M-C, Henrissat B, Coutinho PM, Lombard V, Natvig DO, Lindquist E, Schmutz J, Lucas S, Harris P, Powlowski J, Bellemare A, Taylor D, Butler G, de Vries RP, Allijn IE, van den Brink J, Ushinsky S, Storms R, Powell AJ, Paulsen IT, Elbourne LDH (2011). Comparative genomic analysis of the thermophilic biomass-degrading fungi *Myceliophthora thermophila* and *Thielavia terrestris*. Nat Biotechnol.

[16] Karnaouri A, Topakas E, Antonopoulou I, Christakopoulos P (2014). Genomic insights into the fungal lignocellulolytic system of *Myceliophthora thermophila*. Front Microbiol..

[17] Dashtban M, Schraft H, Qin W (2009). Fungal bioconversion of lignocellulosic residues; opportunities & perspectives. Int J Biol Sci..

[18] Margolles-Clark E, Ilmén M, Penttilä M (1997). Expression patterns of ten hemicellulase genes of the filamentous fungus *Trichoderma reesei* on various carbon sources. J Biotechnol.

[19] Ouyang J, Yan M, Kong D, Xu L (2006). A complete protein pattern of cellulase and hemicellulase genes in the filamentous fungus *Trichoderma reesei*. Biotechnol J.

[20] Hatsch D, Phalip V, Petkovski E, Jeltsch JM (2006). *Fusarium graminearum* on plant cell wall: no fewer than 30 xylanase genes transcribed. Biochem Biophys Res Commun.

[21] Adav SS, Ravindran A, Chao LT, Tan L, Singh S, Sze SK (2011). Proteomic analysis of pH and strains dependent protein secretion of *Trichoderma reesei*. J Proteome Res.

[22] Vinzant TB, Adney WS, Decker SR, Baker JO, Kinter MT, Sherman NE, Fox JW, Himmel ME (2001). Fingerprinting *Trichoderma reesei* hydrolases in a commercial cellulase preparation. Appl Biochem Biotechnol.

[23] Tao L, Schell D, Davis R, Tan E, Elander R, Bratis A (2014). NREL 2012 achievement of ethanol cost targets: biochemical ethanol fermentation via dilute-acid pretreatment and enzymatic hydrolysis of corn stover. NREL/TP-5100-61563.

[24] Alcántara MÁB, Dobruchowska J, Azadi P, García BD, Molina-Heredia FP, Reyes-Sosa FM (2016). Recalcitrant carbohydrates after enzymatic hydrolysis of pretreated lignocellulosic biomass. Biotechnol Biofuels.

[25] Percival Zhang Y-H, Himmel ME, Mielenz JR (2006). Outlook for cellulase improvement: screening and selection strategies. Biotechnol Adv.

[26] Kumar D, Murthy GS, Gupta VK (2016). Enzymatic hydrolysis of cellulose for ethanol production: fundamentals, optimal enzyme ratio, and hydrolysis modeling. New and future developments in microbial biotechnology and bioengineering.

[27] Gusakov AV, Salanovich TN, Antonov AI, Ustinov BB, Okunev ON, Burlingame R, Emalfarb MA, Baez M, Sinitsyn AP (2007). Design of highly efficient cellulase mixtures for enzymatic hydrolysis of cellulose. Biotechnol Bioeng.

[28] Nevalainen H, Peterson R (2014). Making recombinant proteins in filamentous fungi—are we expecting too much?. Front Microbiol..

[29] Karnaouri A, Topakas E, Antonopoulou I, Christakopoulos P (2014). Genomic insights into the fungal lignocellulolytic system of *Myceliophthora thermophila*. Front Microbiol..

[30] Visser H, Joosten V, Punt PJ, Gusakov AV, Olson PT, Joosten R, Bartels J, Visser J, Sinitsyn AP, Emalfarb MA, Verdoes JC, Wery J (2011). Development of a mature fungal technology and production platform for industrial enzymes based on a *Myceliophthora thermophila* isolate, previously known as *Chrysosporium lucknowense* C1. Ind Biotechnol..

[31] Verdoes JC, Punt PJ, Burlingame R, Bartels J, Van Dijk R, Slump E, Meens M, Joosten R, Emalfarb MA (2007). Dedicated vector for efficient library construction and high throughput screening in the hyphal fungus *Chrysosporium lucknowense*. Ind Biotechnol..

[32] Emalfarb MA, Ben-Bassat A, Burlingame RP, Chernoglazov VM, Okounev ON, Olson PT, Sinitsyn AP, Solovjeva IV. Cellulase compositions and methods of use. United States Patent US5811381A, 10 Oct 1996.

[33] UniProt Consortium (2015). UniProt: a hub for protein information. Nucleic Acids Res.

[34] Van den Brink J, Samson RA, Hagen F, Boekhout T, de Vries RP (2012). Phylogeny of the industrial relevant, thermophilic genera *Myceliophthora* and *Corynascus*. Fungal Divers.

[35] Bukhtojarov FE, Ustinov BB, Salanovich TN, Antonov AI, Gusakov AV, Okunev ON, Sinitsyn AP (2004). Cellulase complex of the fungus *Chrysosporium lucknowense*: isolation and characterization of endoglucanases and cellobiohydrolases. Biochem..

[36] Johnson M, Zaretskaya I, Raytselis Y, Merezhuk Y, McGinnis S, Madden TL (2008). NCBI BLAST: a better web interface. Nucleic Acids Res.

[37] Altschul SF, Boguski MS, Gish W, Wootton JC (1994). Issues in searching molecular sequence databases. Nat Genet.

[38] Petersen TN, Brunak S, von Heijne G, Nielsen H (2011). SignalP 4.0: discriminating signal peptides from transmembrane regions. Nat Methods.

[39] Gasteiger E, Hoogland C, Gattiker A, Duvaud S, Wilkins MR, Appel RD, Bairoch A, Walker JM (2005). Protein identification and analysis tools on the ExPASy server. The proteomics protocols handbook.

[40] Parry NJ, Beever DE, Owen E, Vandenberghe I, Van Beeumen J, Bhat MK (2001). Biochemical characterization and mechanism of action of a thermostable beta-glucosidase purified from *Thermoascus aurantiacus*. Biochem J..

[41] Gusakov AV, Sinitsyn AP, Salanovich TN, Bukhtojarov FE, Markov AV, Ustinov BB, Van Zeijl C, Punt P, Burlingame R (2005). Purification, cloning and characterisation of two forms of thermostable and highly active cellobiohydrolase I (Cel7A) produced by the industrial strain of *Chrysosporium lucknowense*. Enzyme Microb Technol.

[42] Sinitsyn AP, Chernoglazov VM, Gusakov AV (1990). Methods of investigation and properties of cellulolytic enzymes. Biotechnol Ser..

[43] McCleary BV (1980). New chromogenic substrates for the assay of alpha-amylase and (1 leads to 4)-beta-d-glucanase. Carbohydr Res.

[44] McCleary BV, Leatham GF, Himmel ME (1991). Comparison of endolytic hydrolases that depolymerize 1,4-beta-d-mannan, 1,5-alpha-l-arabinan, and 1,4-beta-d-galactan. Enzymes in biomass conversion.

[45] Hynes MJ, Corrick CM, King JA (1983). Isolation of genomic clones containing the amdS gene of *Aspergillus nidulans* and their use in the analysis of structural and regulatory mutations. Mol Cell Biol.

[46] Emalfarb MA, Burlingame RP, Olson PT, Sinitsyn AP, Parriche M, Bousson JC, Pynnonen CM, Punt PJ, Van Zeijl CMJ. Transformation system in the field of filamentous fungal hosts: in *Chrysosporium*. United States Patent US6573086, 6 Oct 1998.

[47] Biomass compositional analysis laboratory procedures. National renewable energy laboratory (NREL). http://www.nrel.gov/bioenergy/biomass-compositional-analysis.html. Accessed 15 Jan 2017.

[48] Kristensen JB, Felby C, Jørgensen H (2009). Yield-determining factors in high-solids enzymatic hydrolysis of lignocellulose. Biotechnol Biofuels.

[49] Ishihama Y, Oda Y, Tabata T, Sato T, Nagasu T, Rappsilber J, Mann M (2005). Exponentially modified protein abundance index (emPAI) for estimation of absolute protein amount in proteomics by the number of sequenced peptides per protein. Mol Cell Proteomics.

[50] Rosgaard L, Pedersen S, Langston J, Akerhielm D, Cherry JR, Meyer AS (2007). Evaluation of minimal *Trichoderma reesei* cellulase mixtures on differently pretreated barley straw substrates. Biotechnol Prog.

[51] Beckham GT, Dai Z, Matthews JF, Momany M, Payne CM, Adney WS, Baker SE, Himmel ME (2012). Harnessing glycosylation to improve cellulase activity. Curr Opin Biotech..

[52] Goto M (2007). Protein O-glycosylation in fungi: diverse structures and multiple functions. Biosci Biotechnol Biochem.

[53] Gusakov AV, Antonov AI, Ustinov BB (2008). *N*-glycosylation in *Chrysosporium lucknowense* enzymes. Carbohydr Res.

[54] Teeri TT (1997). Crystalline cellulose degradation: new insight into the function of cellobiohydrolases. Trends Biotechnol.

[55] Wood TM, McCrae SI. Synergism between enzymes involved in the solubilization of native cellulose. In: Brown RD, Jurasek L, editors. Hydrolysis of cellulose: mechanisms of enzymatic and acid catalysis. Adv Chem Ser. 1979. doi: 10.1021/ba-1979-0181.ch010.

[56] Kleman-Leyer KM, Siika-Aho M, Teeri TT, Kirk TK (1996). The cellulases endoglucanase I and cellobiohydrolase II of *Trichoderma reesei* act synergistically to solubilize native cotton cellulose but not to decrease its molecular size. Appl Environ Microbiol.

